# Shared genetics and causal association between plasma levels of SARS‐CoV‐2 entry receptor ACE2 and Alzheimer's disease

**DOI:** 10.1111/cns.14873

**Published:** 2024-07-26

**Authors:** Yan Zhang, Fang Xu, Tao Wang, Zhifa Han, Hong Shang, Kevin Han, Ping Zhu, Shan Gao, Xiaojie Wang, Yanli Xue, Chen Huang, Yan Chen, Guiyou Liu

**Affiliations:** ^1^ Department of Pathology The Affiliated Hospital of Weifang Medical University Weifang China; ^2^ Department of Neurology, Xuanwu Hospital, National Center for Neurological Disorders Capital Medical University Beijing China; ^3^ Academy for Advanced Interdisciplinary Studies Peking University Beijing China; ^4^ Chinese Institute for Brain Research Beijing China; ^5^ Center of Respiratory Medicine, China–Japan Friendship Hospital, National Center for Respiratory Medicine, Institute of Respiratory Medicine Chinese Acadamy of Medical Sciences, National Clinical Research Center for Respiratory Diseases Beijing China; ^6^ Department of Neurology The Fourth Affiliated Hospital of Harbin Medical University Harbin China; ^7^ Department of Statistics Stanford University Stanford California USA; ^8^ Beijing Institute of Brain Disorders, Laboratory of Brain Disorders, Ministry of Science and Technology, Collaborative Innovation Center for Brain Disorders Capital Medical University Beijing China; ^9^ Department of Neurology Shenzhen Qianhai Shekou Free Trade Zone Hospital Shenzhen China; ^10^ School of Biomedical Engineering Capital Medical University Beijing China; ^11^ Dr. Neher's Biophysics Laboratory for Innovative Drug Discovery, State Key Laboratory of Quality Research in Chinese Medicine Macau University of Science and Technology Macao SAR China; ^12^ Department of Epidemiology and Biostatistics, School of Public Health Wannan Medical College Wuhu China; ^13^ Institute of Chronic Disease Prevention and Control Wannan Medical College Wuhu China; ^14^ Beijing Key Laboratory of Hypoxia Translational Medicine, National Engineering Laboratory of Internet Medical Diagnosis and Treatment Technology, Xuanwu Hospital Capital Medical University Beijing China; ^15^ Taishan Vocational College of Nursing Taian China; ^16^ Brain Hospital Shengli Oilfield Central Hospital Dongying China

**Keywords:** *ACE2*, Alzheimer's disease, COVID‐19, expression quantitative trait loci, gene expression, genome‐wide association study, SARS‐CoV‐2

## Abstract

**Background:**

Alzheimer's disease (AD) is the highest risk of COVID‐19 infection, hospitalization, and mortality. However, it remains largely unclear about the link between AD and COVID‐19 outcomes. ACE2 is an entry receptor for SARS‐CoV‐2. Circulating ACE2 is a novel biomarker of death and associated with COVID‐19 outcomes.

**Methods:**

Here, we explored the shared genetics and causal association between AD and plasma ACE2 levels using large‐scale genome‐wide association study, gene expression, expression quantitative trait loci, and high‐throughput plasma proteomic profiling datasets.

**Results:**

We found a significant causal effect of genetically increased circulating ACE2 on increased risk of AD. Cross‐trait association analysis identified 19 shared genetic variants, and three variants rs3104412, rs2395166, and rs3135344 at chromosome 6p21.32 were associated with COVID‐19 infection, hospitalization, and severity. We mapped 19 variants to 117 genes, which were significantly upregulated in lung, spleen, and small intestine, downregulated in brain tissues, and involved in immune system, immune disease, and infectious disease pathways. The plasma proteins corresponding to *LST1*, *AGER*, *TNXB*, and *APOC1* were predominantly associated with COVID‐19 infection, ventilation, and death.

**Conclusion:**

Together, our findings suggest the shared genetics and causal association between AD and plasma ACE2 levels, which may partially explain the link between AD and COVID‐19.

## Graphical abstract

We found the shared genetics and causal association between plasma levels of SARS‐CoV‐2 entry receptor ACE2 and Alzheimer's disease. We found association between plasma proteins corresponding to the shared genes and COVID‐19 outcomes using high‐throughput plasma proteomic profiling.

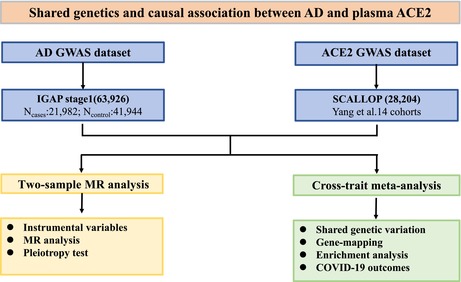

## 1 INTRODUCTION

Severe acute respiratory syndrome coronavirus 2 (SARS‐CoV‐2) had caused coronavirus disease 2019 (COVID‐19), a devastating global pandemic. Evidence indicates that several medical comorbidities, such as chronic obstructive pulmonary disease, asthma, cardiovascular disease (CVD), diabetes, hypertension, and dementias, increase the risk of COVID‐19 infection, hospitalization, and mortality.^1^, ^2^ Importantly, the older population with dementia especially Alzheimer's disease (AD) are facing an unprecedented threat from COVID‐19, and have the highest risk of COVID‐19 infection, hospitalization, and mortality.^1^, ^2^ Meanwhile, COVID‐19 further increases the risk of AD.^3^, ^4^

Angiotensin‐converting enzyme 2 (ACE2) is a protein on the surface of many cell types.^5^ It cuts up the larger protein angiotensinogen into small proteins that then go on to regulate functions in these cells.^5^ Importantly, ACE2 has been identified to be an entry receptor for SARS‐CoV‐2.^5^ In healthy individuals, ACE2 exists mainly in membrane‐bound form, and circulating ACE2 (lacking the transmembrane domain) is relatively low with very low levels of catalytically active ectodomain.^6^, ^7^, ^8^ It is believed that circulating ACE2 is generated from cell‐membrane expressed ACE2, shed by ADAM‐17 and other proteases.^8^ SARS‐CoV‐2 utilizes the catalytic site of full‐length membrane‐bound ACE2 for host cell entry, which is followed by viral internalization together with ACE2 and ACE2 degradation, accelerating the conversion from membrane‐bound ACE2 to circulating ACE2.^5^, ^9^ Therefore, the full‐length membrane‐bound ACE2 levels were markedly reduced and the circulating ACE2 levels were markedly increased upon SARS‐CoV‐2 infection, which have been widely reported in COVID‐19 patients by observational studies.^10^, ^11^ Importantly, high circulating ACE2 associated with increased COVID‐19 severity and mortality, and could be used to predict severity and mortality.^10^, ^11^

In addition to COVID‐19, circulating ACE2 is also a novel biomarker of death and CVD.^12^, ^13^ In patients with CVD, increased circulating ACE2 levels associate with adverse cardiovascular outcomes.^12^ In the general population, high circulating ACE2 levels associate with increased risk of total deaths, incident heart failure, myocardial infarction, stroke, and diabetes independent of age, sex, ancestry, and traditional cardiovascular risk factors.^13^ Compared with the well‐established clinical risk factors (smoking, diabetes, blood pressure, lipids, and body‐mass index), circulating ACE2 is the highest ranked predictor of death, and is also a strong predictor of CVD, including heart failure, stroke, and myocardial infarction.^13^ Importantly, recent findings further support positive genetic association of circulating ACE2 with severe COVID‐19, CVD, asthma, diabetes, and hypertension, as well as causal effect of circulating ACE2 on COVID‐19 infection, hospitalization, and severity.^14^

**FIGURE 1:**
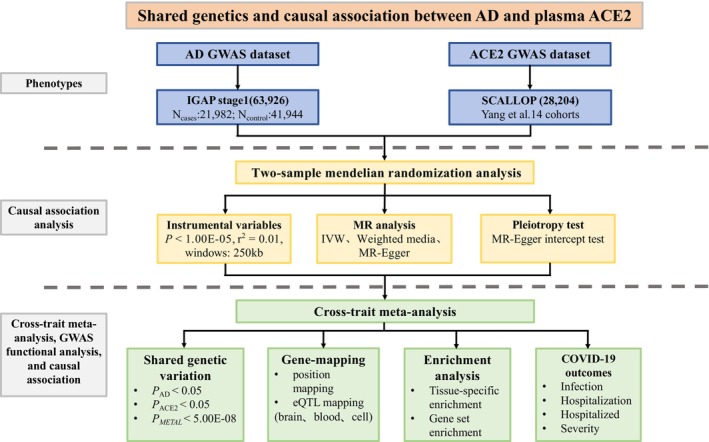
The schematic diagram of the study design in this study.

Collectively, these above findings show that circulating ACE2 shares a genetic basis with COVID‐19 and its established risk factors, and could be a link of COVID‐19 severity and mortality with its established risk factors. Evidence shows that AD pathology might aggravate the consequence of COVID‐19 infection.^15^ However, it currently remains unclear about the genetic association between circulating ACE2 levels and the risk of AD. We consider that there may be shared genetic etiology between circulating ACE2 levels and AD, which may contribute to explain the highest risk of COVID‐19 infection, hospitalization, and mortality in preexisting diagnosis of AD, as well as the increased risk of AD in COVID‐19 patients. Here, we explore the shared genetic etiology between AD and plasma ACE2 levels. In stage 1, we examine the causal association between circulating ACE2 and AD using Mendelian randomization (MR). In stage 2, we identify the shared genetic variants using a cross‐trait association analysis. In stage 3, we map the shared genetic variants to their corresponding genes, and conduct tissue‐specific gene expression analysis, tissue‐specific enrichment analysis, and gene set *enrichment analysis*. In stage 4, we investigate the association of shared genetic variants and their corresponding genes with COVID‐19 outcomes. Figure 1 provides the schematic diagram of the study design in this study.

## 2 MATERIALS AND METHODS

### 2.1 AD GWAS dataset

International Genomics of Alzheimer's Project (IGAP) conducted the largest clinically diagnosed AD genome‐wide association study (GWAS) in 94,437 individuals of European ancestry (35,274 clinical and autopsy‐documented AD and 59,163 controls) from stage 1 (*n* = 63,926), stage 2 (*n* = 18,845), and stage 3A (*n* = 11,666) or stage 3B (*n* = 30,511 from stage 2 + stage 3A).^16^ AD is diagnosed using the same diagnostic criteria including DSM‐III‐R, DSM‐IV, and NINCDS‐ADRDA across the three stages.^16^ There is no clear evidence of cognitive or overall difference across different stages.^16^ In IGAP stage 1, a total of 9,456,058 common variants and 2,024,574 rare variants were imputed and selected for analysis.^16^ In IGAP stage 2, a total of 11,632 variants were further genotyped in 8362 AD cases and 10,483 controls, and were meta‐analyzed with IGAP stage 1.^16^ Here, we selected the IGAP stage 1 for MR analysis as it included the full genetic variants (*n* = 9,456,058), and IGAP stage 1 + stage 2 for cross‐trait association analysis as it included the largest sample size (*n* = 82,777), respectively.

### 2.2 Circulating ACE2 GWAS dataset

In order to understand the genetic basis of the ACE2 protein levels, Yang et al.^14^ performed the largest GWAS meta‐analysis of plasma ACE2 levels measured by Olink platform in 28,204 individuals from 14 cohorts in the SCALLOP consortium (Systematic and Combined Analysis of Olink Proteins). Here, we selected the circulating ACE2 GWAS dataset in both LDSC analysis and cross‐trait association analysis. Yang et al.^14^ only identified 10 independent genome‐wide significant genetic variants including nine in the autosomes and one in the X chromosome, which together explain 4.1% of the phenotypic variance of plasma ACE2 equivalent to about 30% of the heritability. In order to increase more autosomal genetic variants as the potential instrumental variables in MR analysis, we performed a clumping analysis of the plasma ACE2 GWAS dataset to select independent autosomal genetic variants with *p* < 1.00E‐05 using TwoSampleMR v0.5.7 and two key parameters including clumping window 250 kb and clumping *r* ^2^ cutoff 0.01.

### 2.3 Mendelian randomization analysis

We selected inverse‐variance weighted (IVW) as the main MR analysis method, and selected the weighted median and MR‐Egger as the sensitivity analysis methods.^17^, ^18^, ^19^ If all genetic variants are valid instrumental variables, in other words, if there is no evidence of pleiotropy, IVW combines the variant‐specific Wald estimators to get the overall causal estimate.^17^, ^18^, ^19^ If some genetic variants are not valid instrumental variables, in other words, if there is clear evidence of pleiotropy, MR‐Egger could test the potential pleiotropy using MR‐Egger intercept test and accounts for the potential pleiotropy.^17^, ^18^, ^19^ The causal estimate from weighted median is still consistent with the overall causal estimate when at least 50% of the weights come from valid instrumental variables.^17^, ^18^, ^19^ All statistical analyses were performed using the R package “Mendelia nRandomization”^19^ an d R version 4.0.5.

### 2.4 Cross‐trait meta‐analysis

We conducted a cross‐trait meta‐analysis to identify the shared genetic variants in both AD and circulating ACE2 using METAL, which is a popular tool for meta‐analysis of GWAS datasets.^20^ METAL provides two analysis schemes. One scheme, METAL combines the *p* values across different studies by fixed‐effects sample size weighted meta‐analysis taking into account the direction of effect.^20^ The other scheme, METAL combine effect size estimates and standard errors across different studies by fixed‐effects inverse‐variance weighted meta‐analysis.^20^ Here, we selected both analysis schemes to identify the shared genetic variants reaching genome‐wide significance *p* < 5.00E‐08 for meta‐analysis and suggestive trait‐specific significance *p* < 0.05 for AD and circulating ACE2.

### 2.5 Gene mapping

We aim to identify the risk genes corresponding to the shared genetic variants using both positional mapping and expression quantitative trait loci (eQTLs) mapping. For positional mapping, we map the shared genetic variants to the nearest genes using HaploReg v4.1.^21^ For eQTLs mapping, we identify risk genes whose expression might be regulated by the shared genetic variants using multiple publicly available eQTLs datasets from human whole blood, brain tissues, microglial cell, and other human tissues. Here, we selected 49 eQTLs datasets in 49 human tissues from Genotype‐Tissue Expression Project (GTEx version 8),^22^ 1 large‐scale eQTLs meta‐analysis dataset in 1433 brain cortex samples,^23^ 4 eQTLs dataset in 255 primary human microglial samples isolated at autopsy from four different brain regions of 100 individuals with neurodegenerative, neurological, or neuropsychiatric disorders, as well as unaffected controls,^24^ 4 eQTL datasets in whole blood, including 31,684 individuals,^25^ 2765 individuals,^26^ 2116 individuals,^27^ 5257 and individuals.^28^ The statistically significant association is defined to be *p* < 1.00E‐04.

### 2.6 Tissue‐specific gene expression analysis

Using all genes from both positional mapping and eQTLs mapping, we performed a tissue‐specific gene expression analysis by FUMA v1.5.0, which is an online web application to annotate and prioritize genetic associations.^29^ FUMA evaluated gene expression and detected tissue‐specific *enrichment* analysis using expression data from GTEx v8 54 tissue types.^29^ The gene expression value TPM (Transcripts Per Million) is an averaged expression value per tissue type per gene following to winsorization at 50 and log 2 transformation with pseudocount 1.^29^ This kind of averaged expression allows for comparison across tissues and genes.

### 2.7 Tissue‐specific enrichment analysis

Using all genes from both positional mapping and eQTLs mapping, we performed a tissue‐specific enrichment analysis by FUMA v1.5.0.^29^ Tissue‐specific enrichment analysis is tested using the differentially expressed genes (DEGs) defined for each tissue type of each expression dataset.^29^ First, gene expression values were normalized (zero‐mean) following to a log 2 transformation of expression value (TPM).^29^ Second, DEGs were calculated by performing two‐sided *t*‐test for any one of tissue type against all others. Only those genes with Bonferroni corrected *p* value ≤ 0.05 and absolute log fold change ≥0.58 were defined as DEGs.^29^ Third, tissue‐specific enrichment analysis is performed to test if DEGs are overrepresented in any of tissue type against all others using the hypergeometric test. Tissue types with Bonferroni corrected *p* value < 0.05 are defined to be significant enrichment of DEGs.^29^

### 2.8 Gene set enrichment analysis

We performed a gene set *enrichment* analysis of all genes from both positional mapping and eQTLs mapping using WebGestalt (WEB‐based Gene SeT AnaLysis Toolkit), a functional enrichment analysis web tool.^30^ Here, we focused on the KEGG pathways in WebGestalt functional database.^30^ The hypergeometric test was used to detect any overrepresentation of the shared genes among all the genes in a given KEGG pathway.^30^ KEGG pathways with Bonferroni corrected *p* value < 0.05 are defined to be significantly enriched pathways.

### 2.9 Association between genetic variants and COVID‐19 outcomes

We investigated the potential association between the shared genetic variants and COVID‐19 outcomes using large‐scale GWAS datasets from COVID‐19 Human Genetics Initiative, a global initiative to elucidate the role of host genetic factors in susceptibility and severity of the SARS‐CoV‐2 virus pandemic.^31^ We downloaded the GWAS summary statistics from COVID19‐hg GWAS meta‐analyses round 7 for four COVID‐19 outcomes including infection: cases versus population (159,840 cases and 2,782,977 controls), hospitalization: hospitalized cases versus population (44,986 cases and 2,356,386 controls) and hospitalized cases versus not hospitalized cases (16,512 cases and 71,321 controls), and severity: very severe respiratory confirmed cases versus population (18,152 cases and 1,145,546 controls).

### 2.10 Association between shared genes and COVID‐19 outcomes

We evaluated the potential association between plasma proteins corresponding to the shared genes and COVID‐19 outcomes using high‐throughput plasma proteomic profiling from two large independent cohorts including the discovery stage (332 COVID‐19 patients and 150 controls) and replication stage (297 COVID‐19 patients and 76 controls).^32^ Three COVID‐19 outcomes were available in both datasets including COVID‐19 infection (all cases vs. healthy controls that compared all COVID‐19‐positive individuals with samples taken from healthy individuals without COVID‐19), ventilation (cases requiring ventilation vs. cases without ventilation support that compared COVID‐19‐positive individuals whose treatment included ventilation to COVID‐19‐positive individuals who did not), and death (died cases vs. survived cases that contrasted COVID‐19‐related deaths with individuals who had COVID‐19 but did not die of it).^32^ We explored the shared genes using COVID‐19 Proteomics Data and Analytics Browser, which consisted of 1449 proteins associated with any of the three outcomes (841 for infection, 833 for ventilation, and 253 for death).^32^

## 3 RESULTS

### 3.1 Mendelian randomization analysis

We identified 70 independent autosomal genetic variants with *p* < 1.00E‐05 by clumping analysis of the plasma ACE2 GWAS dataset using TwoSampleMR v0.5.7 and two key parameters including clumping window 250 kb and clumping *r* ^2^ cutoff 0.01, as provided in Table S1. Here, we selected these 70 genetic variants as the potential instrumental variables, and extracted their corresponding AD GWAS summary statistics from IGAP stage 1. Using IVW, we identified a significant causal effect of genetically increased circulating ACE2 level on increased risk of AD (OR = 1.12, 95% CI: 1.05–1.21, *p* = 0.001). Importantly, MR estimates from weighted median (OR = 1.10, 95% CI: 0.97–1.23, *p* = 0.134) and MR‐Egger (OR = 1.10, 95% CI: 0.95–1.29, *p* = 0.203) were consistent with IVW estimate in terms of direction and magnitude although lack of statistically significance. Meanwhile, MR‐Egger intercept test indicates no evidence of pleiotropy with intercept = 0.001 and *p* = 0.793.

### 3.2 Cross‐trait meta‐analysis

Using fixed‐effects sample size weighted meta‐analysis, we found 19 genetic variants that were associated with both AD and circulating ACE2 at the genome‐wide significance *p* < 5.00E‐08 for the cross‐trait meta‐analysis and suggestive trait‐specific significance *p* < 0.05 for AD and circulating ACE2 with the same directions of effect sizes (Table 1); 4, 1, 1, and 13 genetic variants are located at chromosome 6p21.32, 8p21.2‐p21.1, 17p13.2, and 19q13.32, respectively. These genetic variants are in linkage disequilibrium with each other. Using fixed‐effects inverse‐variance weighted meta‐analysis, these 19 genetic variants were further verified, and three genetic variants rs9269853, rs2395166, and rs10415074 reached the genome‐wide significance *p* < 5.00E‐08, as provided in Table 1.

**TABLE 1:** Shared genetic variants from cross‐trait meta‐analysis of AD and circulating ACE2 with *p* < 5.00E‐08 and single trait *p* < 0.05.

SNP	Chr	Position	EA	NEA	Z score	*p* Value	β	SE	*p* Value	Direction	Gene
rs2395166	6	32388275	t	c	−6.543	6.02E‐11	−0.0428	0.0076	1.51E‐08	−	8.8 kb 5′ of *BTNL2*
rs3135344	6	32395036	t	c	−5.987	2.14E‐09	−0.0412	0.0079	1.70E‐07	−	13 kb 5′ of *HLA‐DRA*
rs9269853	6	32550322	a	c	5.969	2.38E‐09	0.0887	0.0149	2.63E‐09	++	*HLA‐DRB1*
rs3104412	6	32585967	a	g	5.799	6.68E‐09	0.0399	0.0075	8.90E‐08	++	10 kb 5′ of *HLA‐DQA1*
rs17466060	8	27422740	a	g	5.539	3.04E‐08	0.0353	0.0073	1.50E‐06	++	20 kb 3′ of *EPHX2*
rs12150370	17	4777634	t	c	−5.464	4.66E‐08	−0.0629	0.0119	1.16E‐07	−	*MINK1*
rs1551891	19	45231821	a	g	−6.577	4.80E‐11	−0.0738	0.0142	2.00E‐07	−	2.5 kb 5′ of *snoZ6*
rs62117161	19	45233385	a	g	6.645	3.02E‐11	0.0734	0.0143	2.66E‐07	++	4.1 kb 5′ of *snoZ6*
rs62117162	19	45239536	a	c	−6.115	9.68E‐10	−0.0682	0.0142	1.46E‐06	−	10 kb 5′ of *snoZ6*
rs4803750	19	45247627	a	g	6.635	3.24E‐11	0.0762	0.0154	7.13E‐07	++	3.3 kb 5′ of *BCL3*
rs10401176	19	45253491	t	c	−6.683	2.34E‐11	−0.0608	0.0119	3.26E‐07	−	*BCL3*
rs10415074	19	45341759	c	g	−11.81	3.47E‐32	−0.1351	0.0127	2.99E‐26	−	7.6 kb 5′ of *PVRL2*
rs141739979	19	45374983	t	g	−5.974	2.32E‐09	−0.2206	0.0435	4.02E‐07	−	*PVRL2*
rs113345881	19	45431636	a	g	−5.928	3.08E‐09	−0.0597	0.0133	6.99E‐06	−	9 kb 3′ of *APOC1*
rs112871012	19	45431897	a	g	5.622	1.88E‐08	0.0567	0.0133	1.90E‐05	++	9.3 kb 3′ of *APOC1*
rs7259350	19	45432278	t	c	−5.829	5.58E‐09	−0.0582	0.0133	1.15E‐05	−	9.7 kb 3′ of *APOC1*
rs7259004	19	45432557	c	g	5.639	1.71E‐08	0.0578	0.0132	1.22E‐05	++	10 kb 3′ of *APOC1*
rs143674704	19	45458466	a	g	−6.802	1.03E‐11	−0.146	0.0296	8.36E‐07	−	*CLPTM1*
rs76271385	19	45612862	a	g	5.525	3.29E‐08	0.1865	0.0419	8.52E‐06	++	*PPP1R37*

*Note*: The position is based on GRCh37/hg19.

Abbreviations: Chr, chromosome; EA, effect allele; NEA, non‐effect allele.

### 3.3 Gene mapping using positional mapping

Using HaploReg v4.1, we mapped these 19 genetic variants to their corresponding nearby genes as provided in Table 1. Four genetic variants at chromosome 6p21.32 are mapped to *BTNL2*, *HLA‐DRA*, *HLA‐DRB1*, and *HLA‐DQA1*. Genetic variant rs17466060 located at chromosome 8p21.2‐p21.1 is mapped to 20 kb 3′ of *EPHX2*. Genetic variant rs12150370 located at chromosome 17p13.2 is mapped to *MINK1*. Thirteen genetic variants located at chromosome 19q13.32 are mapped to *snoZ6*, *BCL3*, *PVRL2*, *APOC1*, *CLPTM1*, and *PPP1R37* (Table 1).

### 3.4 Gene mapping using eQTLs analysis

eQTLs analysis not only confirms those findings from positional mapping, but also highlights some novel findings. Four genetic variants at chromosome 6p21.32 are associated with the expression of 65 genes including *HLA‐DPB1*, *HLA‐DMA*, *HLA‐DMB*, *HLA‐DOB*, *HLA‐DPA1*, *HLA‐DPB2*, *HLA‐DQA1*, *HLA‐DQA2*, *HLA‐DQB1*, *HLA‐DQB1‐AS1*, *HLA‐DQB2*, *HLA‐DRA*, *HLA‐DRB1*, *HLA‐DRB4*, *HLA‐DRB5*, *HLA‐DRB6*, *HLA‐DRB9*, *AGER*, *ATF6B*, *PSMB9*, *TNXB*, *C2*, and *LST1*. rs17466060 variant located at chromosome 8p21.2‐p21.1 was associated with the expression of three genes including *EPHX2*, *CLU*, and *GULOP*. rs12150370 variant located at chromosome 17p13.2 was associated with the expression of 33 genes such as *CAMTA2*, *CHRNE*, *INCA1*, *MINK1*, *PLD2*, *CXCL16*, *KIF1C*, *SCIMP*, *GP1BA*, *ENO3*, and *ZNF232*. Thirteen genetic variants located at chromosome 19q13.32 associated with the expression of 12 genes such as *BCL3*, *PVR*, *PVRL2*, *NKPD1*, *MARK4*, *CEACAM19*, *NECTIN2*, *BCAM*, *APOC1P1*, and *CKM*. Here, we have provided more detailed eQTLs analysis results in Tables S2–S8.

### 3.5 Tissue‐specific gene expression analysis

**FIGURE 2:**
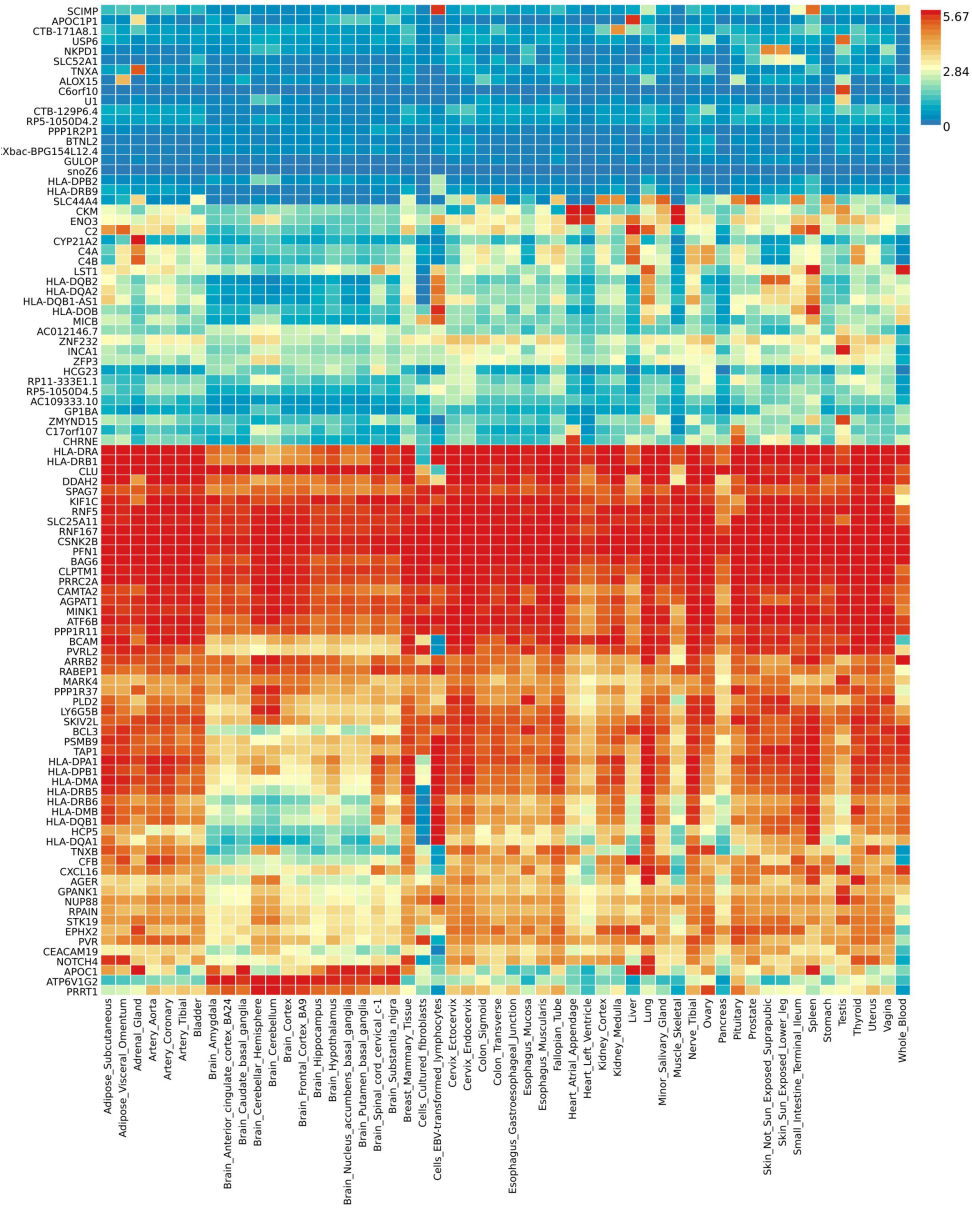
Heat map of tissue‐specific gene expression of genes corresponding to shared genetic variants from cross‐trait meta‐analysis of AD and circulating ACE2. The heat map is plotted using FUMA v1.5.0 and gene expression data from GTEx v8 54 tissue types. The heat map was ordered by both gene and tissue clustering. Darker red represent higher expression of that gene compared to darker blue color across genes and tissues.

We got a total of 117 unique genes using positional mapping and/or eQTLs mapping. A total of 102 and 94 genes were recognized with recognized Ensembl ID and Ensembl ID in FUMA, respectively. Tissue‐specific gene expression results are provided in Figure 2 as a gene expression heat map, which is clustered by both genes and tissues. The results showed that some genes were highly expressed across GTEx v8 54 tissue types, such as *HLA‐DRA*, *HLA‐DRB1*, *CLU*, *KIF1C*, *BAG6*, *CLPTM1*, *MINK1*, and *ATF6B*. Meanwhile, some genes were highly expressed specifically in some tissues, such as *LST1* and *AGER*. *LST1* only showed high expression levels in whole blood, spleen, and lung. *AGER* just showed high expression levels in lung and thyroid.

### 3.6 Tissue‐specific enrichment analysis

**FIGURE 3:**
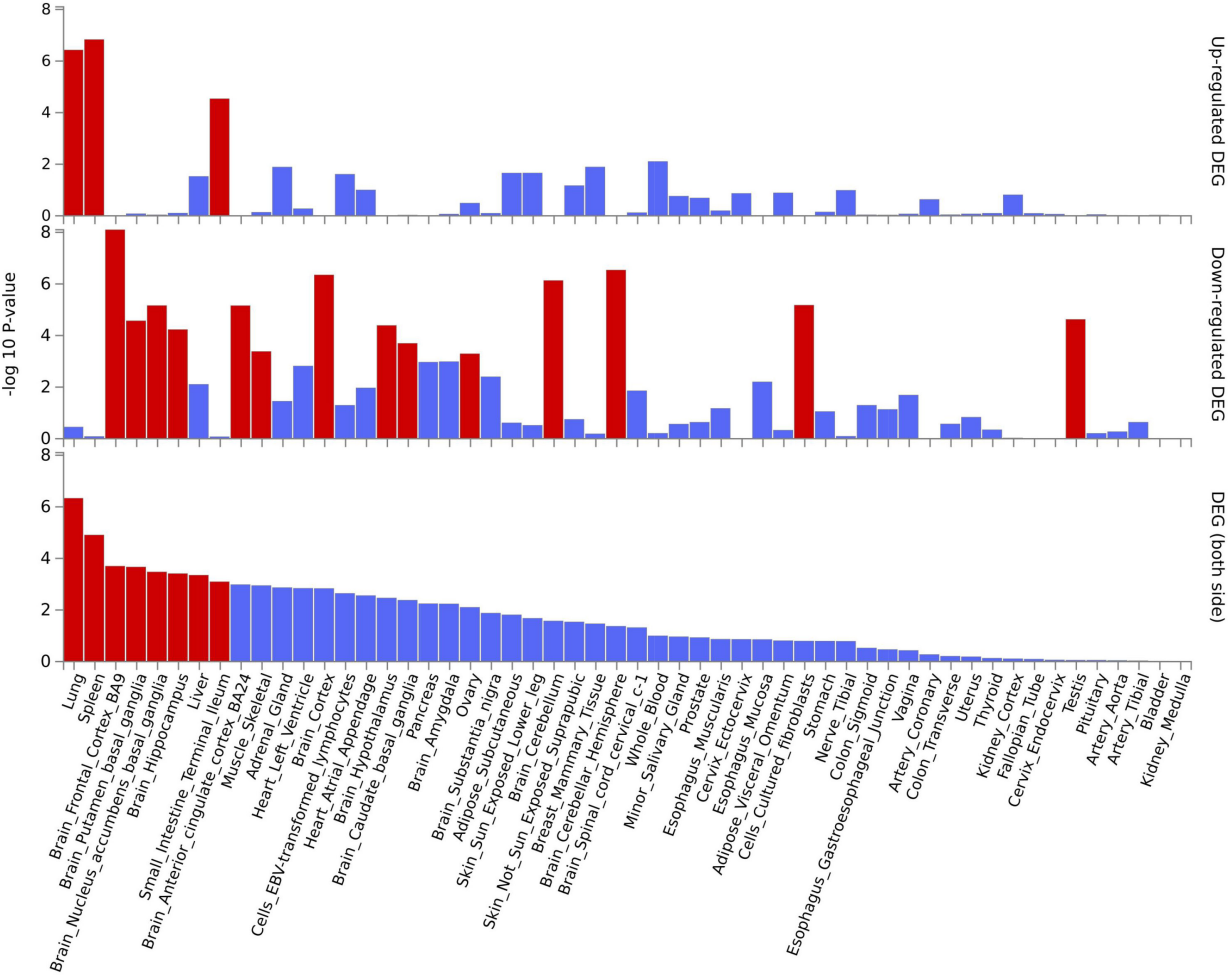
Tissue‐specific gene expression enrichment analysis of differentially expressed genes across GTEx v8 54 tissue types. Enrichment of differentially expressed genes was identified using FUMA v1.5.0 and gene expression data from GTEx v8 54 tissue types. Tissue types with Bonferroni corrected *p* value ≤ 0.05 are defined to be significant enrichment of differentially expressed genes, and are highlighted in red.

Using GTEx v8 54 tissue ty pes, DEGs are significantly enriched in lung, spleen, and small intestine with Bonferroni corrected *p* value < 0.05, which are highlighted in red as provided in Figure 3. Interestingly, subgroup analysis using the upregulated DEGs and downregulated DEGs further supports that only upregulated DEGs are significantly enriched in lung, spleen, and small intestine with Bonferroni corrected *p* value < 0.05. Meanwhile, we found that downregulated DEGs were significantly enriched in multiple brain tissues including putamen (basal ganglia), frontal Cortex (BA9), hippocampus, nucleus accumbens (basal ganglia), anterior cingulate cortex (BA24), cortex, cerebellum, and cerebellar hemisphere. Here, we have provided more detailed results from the tissue specificity test in Table S9.

### 3.7 Gene set enrichment analysis

**FIGURE 4:**
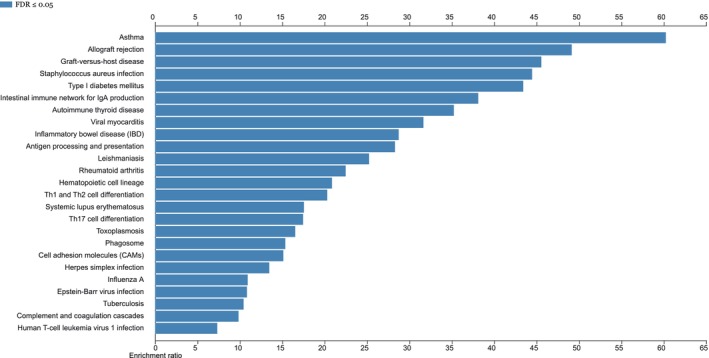
Gene set enrichment analysis of genes corresponding to shared genetic variants from cross‐trait meta‐analysis of AD and circulating ACE2. Gene set enrichment analysis was performed using WebGestalt (WEB‐based GEne SeT AnaLysis Toolkit). KEGG pathways with Bonferroni corrected *p* value < 0.05 are defined to be significantly enriched pathways.

We identified 25 significantly enriched pathways as provided in Figure 4. KEGG pathway classifications shows that most of these pathway are associated with immune system and immune diseases, such as autoimmune thyroid disease, intestinal immune network for IgA production, type I diabetes mellitus, graft‐versus‐host disease, allograft rejection, and asthma. Meanwhile, other pathways are associated with infectious diseases, including staphylococcus aureus infection, leishmaniasis, herpes simplex infection, toxoplasmosis, Epstein–Barr virus infection, Influenza A, Tuberculosis, and Human T‐cell leukemia virus 1 infection. Here, we have provided more detailed results from gene set enrichment analysis in Table S10.

### 3.8 Association between shared genetic variants and COVID‐19 outcomes

Using the GWAS summary statistics from COVID19‐hg GWAS meta‐analyses round 7, we found suggestive (*p* value < 0.05) or statistically significant (Bonferroni corrected *p* value < 0.05/(19*4) = 6.58E‐04, as 19 genetic variants and four COVID‐19 outcomes) association of three genetic variants at chromosome 6p21.32 (rs3104412, rs2395166, and rs3135344) and two genetic variants at 19q13.32 (141,739,979, rs143674704) with COVID‐19 outcomes, as provided in Table 2. In brief, three genetic variants including rs3104412, rs141739979, and rs143674704 were associated with COVID‐19 infection, three genetic variants including rs2395166, rs3135344, and rs3104412 were associated with COVID‐19 hospitalization, two genetic variants including rs3135344 and rs3104412 were associated with COVID‐19 severity, as provided in Table 2. Importantly, the directions of the effect alleles from rs3104412, rs2395166, and rs3135344 are consistent across AD, circulating ACE2, and COVID‐19 outcomes. Here, we provided all association results in Table S11.

**TABLE 2:** Association between shared genetic variants and COVID‐19 outcomes with *p* < 0.05.

COVID‐19 outcomes	SNP	Chr	Position	EA	NEA	β	SE	*p* Value
COVID‐19 versus population	rs3104412	6	32585967	A	G	8.31E‐03	4.21E‐03	4.84E‐02
COVID‐19 versus population	rs141739979	19	45374983	T	G	5.51E‐02	2.68E‐02	3.94E‐02
COVID‐19 versus population	rs143674704	19	45458466	A	G	3.52E‐02	1.73E‐02	4.22E‐02
Hospitalized COVID‐19 versus population	rs2395166	6	32388275	T	C	−1.78E‐02	8.81E‐03	4.34E‐02
Hospitalized COVID‐19 versus population	rs3135344	6	32395036	T	C	−3.53E‐02	9.70E‐03	2.68E‐04
Hospitalized COVID‐19 versus population	rs3104412	6	32585967	A	G	3.01E‐02	8.45E‐03	3.65E‐04
Hospitalized COVID‐19 versus not hospitalized COVID‐19	rs3135344	6	32395036	T	C	−4.97E‐02	1.99E‐02	1.26E‐02
Very severe respiratory confirmed COVID‐19 versus population	rs3135344	6	32395036	T	C	−4.91E‐02	1.48E‐02	8.78E‐04
Very severe respiratory confirmed COVID‐19 versus population	rs3104412	6	32585967	A	G	5.29E‐02	1.28E‐02	3.47E‐05

*Note*: The position is based on GRCh37/hg19. We define the suggestive association using *p* value < 0.05, and statistically significant using Bonferroni corrected *p* value < 0.05/12 = 4.17E‐03, as three genetic variants and four COVID‐19 outcomes.

Abbreviations: Chr, chromosome; EA, effect allele; NEA, non‐effect allele.

### 3.9 Association between shared genes and COVID‐19 outcomes

**FIGURE 5:**
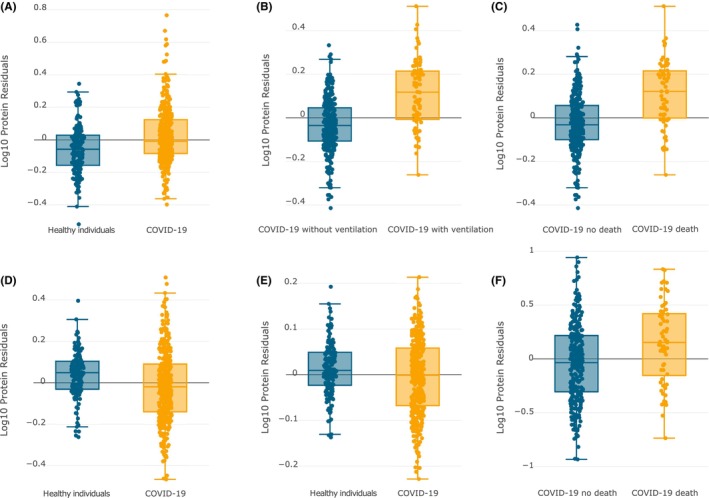
Abundance distributions of plasma proteins corresponding to *LST1*, *AGER*, *TNXB*, and *APOC1* in different COVID‐19 outcomes. Box plots were plotted using COVID‐19 Proteomics Data and Analytics Browser. (A) Abundance distributions of plasma proteins corresponding to *LST1* in COVID‐19 infection (all cases vs. healthy controls) with *p* value = 3.72E‐48; (B) Abundance distributions of plasma proteins corresponding to *LST1* in COVID‐19 ventilation (cases requiring ventilation vs. cases without ventilation support) with *p* value = 2.82E‐31; (C) Abundance distributions of plasma proteins corresponding to *LST1* in COVID‐19 death (died cases vs. survived cases) with *p* value = 2.40E‐16; (D) Abundance distributions of plasma proteins corresponding to *TNXB* in COVID‐19 infection (all cases vs. healthy controls) with *p* value = 1.23E‐45; (E) Abundance distributions of plasma proteins corresponding to *APOC1* in COVID‐19 infection (all cases vs. healthy controls) with *p* value = 7.94E‐29; (F) Abundance distributions of plasma proteins corresponding to *AGER* in COVID‐19 death (died cases vs. survived cases) with *p* value = 5.75E‐06.

Using high‐throughput plasma proteomic profiling from the meta‐analysis of two large independent cohorts including discovery cohorts (332 COVID‐19 patients and 150 controls) and replication cohorts (297 COVID‐19 patients and 76 controls),^32^ we found association between plasma proteins corresponding to the shared genes and COVID‐19 outcomes, as provided in Table 2. Using the GWAS summary statistics from COVID19‐hg GWAS meta‐analyses round 7, we found suggestive association (*p* value < 0.05) or statistically significant association (Bonferroni corrected *p* value < 0.05/(117*3) = 1.42E‐04, as 117 genes and 3 COVID‐19 outcomes), as provided in Table 3. Fourteen genes are associated with COVID‐19 infection with *p* < 0.05, and *LST1* (*p* = 3.72E‐48), *TNXB* (*p* = 1.23E‐45), *APOC1* (*p* = 7.94E‐29), *C2* (*p* = 9.77E‐16), and *SCIMP* (*p* = 3.47E‐10) are the top five significant signals. Eleven genes are associated with COVID‐19 ventilation with *p* < 0.05, and *LST1* (*p* = 2.82E‐31), *PSMB9* (*p* = 1.26E‐13), *KIF1C* (*p* = 1.17E‐10), *SCIMP* (*p* = 4.07E‐07), and *AGER* (*p* = 3.09E‐05) are the top five significant signals. Eight genes are associated with COVID‐19 death with *p* < 0.05, and *LST1* (*p* = 2.40E‐16), *KIF1C* (*p* = 7.76E‐09), *PSMB9* (*p* = 3.63E‐08), *AGER* (*p* = 5.75E‐06), and *BCAM* (*p* = 5.62E‐04) are the top five significant signals. *LST1* not only is the most significant signal that associates with COVID‐19 infection, ventilation, and death, but also shows the largest effects on COVID‐19 infection (β = 0.3451), ventilation (β = 0.176), and larger effect on death (β = 0.1524). *AGER* indicates the largest effect on COVID‐19 death (β = 0.1876). Meanwhile, *TNXB* and *APOC1* also have larger effects on COVID‐19 infection with β = −0.2418 and β = −0.1398, respectively. Here, we have provided more detailed results in Table 3. Figure 5 provides the abundance distributions of plasma proteins corresponding to *LST1*, *AGER*, *TNXB*, and *APOC1* in different COVID‐19 outcomes.

**TABLE 3:** Association between shared genes and COVID‐19 outcomes.

Region	Gene	COVID‐19 infection		COVID‐19 ventilation		COVID‐19 death	
		β	*p* Value	β	*p* Value	β	*p* Value
6p21.32	*AGER*	0.0884	2.14E‐02	0.1361	3.09E‐05	0.1876	5.75E‐06
	*ATF6B*	−0.0193	2.24E‐01	−0.0154	2.69E‐01	−0.0169	3.31E‐01
	*C2*	0.0982	9.77E‐16	0.0085	3.80E‐01	0.0063	5.89E‐01
	*CSNK2B*	0.0414	2.24E‐05	−0.003	6.92E‐01	−0.0047	6.31E‐01
	*HLA‐DQA2*	−0.0396	3.31E‐05	0.0114	7.41E‐02	0.0079	3.02E‐01
	*LST1*	0.3451	3.72E‐48	0.176	2.82E‐31	0.1524	2.40E‐16
	*PSMB9*	−0.0222	1.00E‐01	−0.0768	1.26E‐13	−0.0714	3.63E‐08
	*TNXB*	−0.2418	1.23E‐45	−0.0327	2.40E‐02	−0.0225	2.19E‐01
8p21.2‐p21.1	*CLU*	0.0541	1.62E‐09	−0.004	6.03E‐01	−0.0133	1.74E‐01
17p13.2	*CXCL16*	−0.0548	2.14E‐04	0.048	1.58E‐04	0.0433	1.00E‐02
	*ENO3*	0.0818	6.17E‐03	0.0673	5.25E‐03	0.0524	7.76E‐02
	*GP1BA*	−0.0225	5.75E‐02	−0.0187	3.09E‐02	0.0033	7.59E‐01
	*KIF1C*	0.0031	7.59E‐01	0.0529	1.17E‐10	0.0588	7.76E‐09
	*SCIMP*	−0.0678	3.47E‐10	−0.0312	4.07E‐07	−0.0182	1.17E‐02
19q13.32	*BCAM*	0.033	3.47E‐03	0.0201	9.55E‐03	0.0338	5.62E‐04
	*CEACAM19*	−0.0353	1.51E‐04	−0.0036	5.50E‐01	−0.0068	3.31E‐01
	*NECTIN2*	−0.0106	3.98E‐01	0.0293	5.01E‐04	0.0337	8.13E‐04
	*PVR*	−0.0722	3.02E‐06	0.0115	1.86E‐01	0.0026	7.94E‐01
	*APOC1*	−0.1398	7.94E‐29	−0.0284	2.09E‐03	−0.0216	5.01E‐02

*Note*: We define the suggestive association using *p* value < 0.05, and statistically significant using Bonferroni corrected *p* value < 0.05/(117*3) = 1.42E‐04, as 117 genes and three COVID‐19 outcomes.

## 4 DISCUSSION

Until now, growing evidence showed the involvement of ACE2 and related genes in the serum or plasma of AD or other diseases related to aging. AD cases had decreased ACE2 activity in the serum compared with normal control individuals.^33^ Singh et al.^34^ found the reduced levels of soluble ACE2 in plasma in stroke‐operated mice compared to sham mice. Parkinson's disease (PD) patients had significantly higher serum levels of ACE2 autoantibodies than controls.^35^

Here, we explored the shared genetic etiology between AD and plasma ACE2 levels by a comprehensive analysis. In stage 1, we conducted a causal association analysis. We found a significant causal effect of genetically increased circulating ACE2 level on increased risk of AD. Our current finding is consistent with recent study evaluating the genetic association between circulating ACE2 and other COVID‐19 risk factors in both magnitude and direction.^14^ Yang et al.^14^ have identified significant positive genetic relations between circulating ACE2 and several COVID‐19 medical comorbidities including asthma, diabetes, coronary artery disease, hypertension, and other vascular disease‐related phenotypes. Yang et al. also investigated the genetic relation between circulating ACE2 and AD using AD GWAS dataset from IGAP 2013, including 17,008 AD and 37,154 controls.^36^ However, they did not identify any significant genetic relation between circulating ACE2 and AD (*rg* = 0.0563, *rg*_SE = 0.0998, *p* = 0.573). Here, we used the largest AD GWAS in 94,437 individuals of European ancestry.^16^ Therefore, the large‐scale AD GWAS dataset may contribute to identify more significant positive genetic relation.

In stage 2, we performed a cross‐trait association analysis, and found 19 genetic variants that were significantly associated with both AD and circulating ACE2 at the genome‐wide significance *p* < 5.00E‐08 at chromosome 6p21.32, 8p21.2‐p21.1, 17p13.2, and 19q13.32. In stage 3, we mapped these 19 genetic variants to 117 corresponding genes using positional mapping and eQTLs analysis. Interestingly, growing evidence supports our current findings that these genes are associated with AD and/or COVID‐19. At chromosome 6p21.32, *HLA‐DRA*, *HLA‐DRB1*, and *HLA‐DQA1* are also identified to be AD risk genes.^16^ A gene prioritization approach highlights *HLA‐DRB1*, *HLA‐DRA*, *HLA‐DQA1*, *HLA‐DPA1*, and *HLA‐DRB5* to be the top candidate genes among 46 genes in the MHC locus.^16^ Cell specific peripheral immune responses indicate that *HLA‐DQA1*, *HLA‐DRB5*, and *HLA‐DPB1* are the most predictive of survival in CD16 monocytes from critical COVID‐19 patients.^37^

At chromosome 8p21.2‐p21.1, *EPHX2* encodes soluble epoxide hydrolase (sEH), a key enzyme for epoxyeicosatrienoic acid (EET) signaling.^38^, ^39^ sEH inhibition or *Ephx2* deletion delays AD progression and alleviates AD pathology in mouse models of AD.^38^, ^39^ Evidence from 50 COVID‐19 patients and 94 age‐ and sex‐matched controls shows that SARS‐CoV‐2 serum had significantly increased sEH activity compared to age‐ and sex‐matched SARS‐CoV‐2‐negative group.^40^ At chromosome 19q13.32, *BCL3* is a nuclear member of the inhibitor of NF‐κB family, which regulates the NF‐κB signaling pathway.^41^ Gene‐based test of AD GWAS datasets have identified BCL3 to be an AD susceptibility gene.^42^ Differential gene expression analysis revealed a downregulation of BCL3 in COVID‐19 patients compared to controls in lung, liver, kidney, and heart tissues.^43^, ^44^

Tissue‐specific gene expression analysis showed that some genes were highly expressed across GTEx v8 54 tissue types, and others were highly expressed specifically in specific tissues, such as *LST1* and *AGER*. Tissue‐specific enrichment analysis suggested that these genes were significantly upregulated in lung, spleen, and small intestine, and downregulated in brain tissues. Gene set *enrichment analysis highlighted* significantly enriched pathways involved in immune system, immune diseases, and infectious diseases. Our findings are in line with the pathology observed in post‐mortem tissues obtained from COVID‐19 patients. COVID‐19 causes multi‐organ dysfunction, and predominantly affects the lung, and also harms other body organs including spleen, small intestine, heart, gut, liver, kidneys, and brain.^45^, ^46^

In stage 4, we investigated the association of shared genetic variants and their corresponding genes with COVID‐19 outcomes. We identified three genetic variants rs3104412, rs2395166, and rs3135344 at chromosome 6p21.32 that associated with COVID‐19 infection, hospitalization, and severity. Importantly, these three genetic variants had the same directions of the effect alleles across AD, circulating ACE2, and COVID‐19 outcomes. Meanwhile, we found that the plasma proteins corresponding to *LST1*, *AGER*, *TNXB*, and *APOC1* were predominantly associated with COVID‐19 infection, ventilation, and death. Interestingly, recent findings support the involvement of *LST1*, *AGER*, *TNXB*, and *APOC1* in COVID‐19. Interestingly, recent findings support the involvement of *LST1*, *AGER*, *TNXB*, and *APOC1* in COVID‐19. A large‐scale genome‐wide analysis has identified *LST1* to be a COVID‐19 locus and a potential effector gene.^47^ Single‐cell RNA‐Seq datasets in COVID‐19 patients suggested that *LST1* may play a role in the effect of Angiotensin II receptor blocker on COVID‐19‐related mortality.^48^ Therefore, LST1 not only contributes to predict the COVID‐19 outcomes, but also may be a potential COVID‐19 treatment target. *AGER* is also named *RAGE*, and its plasma protein level was identified to be significantly upregulated in ICU COVID‐19 patients compared to controls.^49^ High level of soluble RAGE is associated with a greater risk of mortality in COVID‐19 patients treated with dexamethasone,^50^ and is considered to be a biomarker of COVID‐19 disease severity and indicator of the need for mechanical ventilation, acute respiratory distress syndrome and mortality.^51^

Our current study still has some limitations. First, it is important to check the results of ACE2 and related genes in the serum in three stages of AD (early stage, middle stage, and late stage) and normal controls. However, there are no large‐scale publicly available serum data from normal controls and AD including early stage, middle stage, and late stage. We will further evaluate the ACE2 and related genes in the serum when relevant data is publicly available in future. Second, in addition to ACE2, there are some other factors increase the risk of COVID‐19. Evidence shows that several genes contribute to viral entry into the cell and viral persistence including *TMPRSS2*, *TPCN2*, *TMPRSS4*, *NRP1*, *CTSL*, *CD147*, *DPP4*, and *TMEM106B*.^5^, ^52^

Together, our findings suggest the shared genetic etiology between plasma ACE2 levels and AD, and plasma ACE2 levels may partially explain the link between AD and COVID‐19. Our findings have potential clinical implications. On the one hand, AD patients with plasma ACE2 levels may have increased risk of COVID‐19 infection, hospitalization, and mortality, and assessment of plasma ACE2 levels may be a means of identifying AD patients at high risk for adverse COVID‐19 outcomes. On the other hand, COVID‐19 patients with plasma ACE2 levels may have increased risk of AD, and assessment of plasma ACE2 levels may be a means of identifying COVID‐19 patients at high risk of AD.

## AUTHOR CONTRIBUTIONS

GYL, YZ, and YC conceived and initiated the project. GYL, YZ, TW, and ZFH analyzed the data, and wrote the first draft of the manuscript. All authors contributed to the interpretation of the results and critical revision of the manuscript for important intellectual content and approved the final version of the manuscript.

## FUNDING INFORMATION

This work was supported by funding from the National Natural Science Foundation of China (Grant Nos 82071212, and 81901181), Natural Science Foundation of Heilongjiang Province (Grant No. LH2019H076), Beijing Natural Science Funds for Distinguished Young Scholar (Grant No. JQ21022), the Mathematical Tianyuan Fund of the National Natural Science Foundation of China (Grant No. 12026414), and Beijing Ten Thousand Talents Project (Grant No. 2020A15). This work was also partially supported by funding from the Science and Technology Beijing One Hundred Leading Talent Training Project (Z141107001514006), the Beijing Municipal Administration of Hospitals' Mission Plan (SML20150802), the Funds of Academic Promotion Programme of Shandong First Medical University & Shandong Academy of Medical Sciences (Nos 2019QL016, 2019PT007).

## CONFLICT OF INTEREST STATEMENT

The authors declare no conflict of interest.

## CONSENT STATEMENT

This article contains human participants collected by several studies performed by previous studies. All participants gave informed consent in all the corresponding original studies. Here, our study is based on the publicly available datasets, and not the individual‐level data. Therefore, consent was not necessary.

## Supporting information


Tables S1–S11:

## Data Availability

All relevant data are within the paper. The authors confirm that all data underlying the findings are either fully available without restriction through consortia websites, or may be made available from consortia upon request. ACE2 GWAS summary statistics: https://figshare.com/articles/dataset/SCALLOP_ACE2_GWAS_Summary_Statistics/19189307. AD GWAS summary statistics: https://www.niagads.org/datasets/ng00075. COVID19‐hg GWAS meta‐analyses round 7: https://www.covid19hg.org/results/r7/. COVID‐19 Proteomics Data and Analytics Browser: https://covid.proteomics.wustl.edu/. GTEx: https://www.gtexportal.org/. eQTLGen Consortium: https://www.eqtlgen.org/.
